# Biotic Control of Skeletal Growth by Scleractinian Corals in Aragonite–Calcite Seas

**DOI:** 10.1371/journal.pone.0091021

**Published:** 2014-03-07

**Authors:** Tomihiko Higuchi, Hiroyuki Fujimura, Ikuko Yuyama, Saki Harii, Sylvain Agostini, Tamotsu Oomori

**Affiliations:** 1 Graduate School of Science and Technology, Shizuoka University, Suruga-ku, Shizuoka, Japan; 2 Department of Chemistry, Biology and Marine Science, University of the Ryukyus, Nishihara, Okinawa, Japan; 3 Sesoko Station, Tropical Biosphere Research Center, University of the Ryukyus, Motobu, Okinawa, Japan; 4 Shimoda Marine Research Center, University of Tsukuba, Shimoda, Shizuoka, Japan; Heriot-Watt University, United Kingdom

## Abstract

Modern scleractinian coral skeletons are commonly composed of aragonite, the orthorhombic form of CaCO_3_. Under certain conditions, modern corals produce calcite as a secondary precipitate to fill pore space. However, coral construction of primary skeletons from calcite has yet to be demonstrated. We report a calcitic primary skeleton produced by the modern scleractinian coral *Acropora tenuis*. When uncalcified juveniles were incubated from the larval stage in seawater with low *m*Mg/Ca levels, the juveniles constructed calcitic crystals in parts of the primary skeleton such as the septa; the deposits were observable under Raman microscopy. Using scanning electron microscopy, we observed different crystal morphologies of aragonite and calcite in a single juvenile skeleton. Quantitative analysis using X-ray diffraction showed that the majority of the skeleton was composed of aragonite even though we had exposed the juveniles to manipulated seawater before their initial crystal nucleation and growth processes. Our results indicate that the modern scleractinian coral *Acropora* mainly produces aragonite skeletons in both aragonite and calcite seas, but also has the ability to use calcite for part of its skeletal growth when incubated in calcite seas.

## Introduction

In recent decades, marine biogenic skeletal formation and its response to global environmental change have become major research topics. The molar magnesium/calcium (Mg/Ca) ratio (*m*Mg/Ca) of seawater has varied between 0.5 and 5.3 through the current Phanerozoic eon [1]. During this period, three episodes of aragonite-facilitating conditions (high *m*Mg/Ca) have occurred, along with two episodes of calcite-facilitating conditions (low *m*Mg/Ca), which are known as aragonite and calcite seas, respectively [2]. Marine calcifying organisms that produce sediments and build reefs generally have skeletons and shells composed of either aragonite or calcite, and long-term changes in the *m*Mg/Ca of seawater tend to correspond to changes in the prevailing mineralogy of these organisms [3], [4]. Fossil records show that the reign of scleractinian corals was interrupted during the mid-Cretaceous Period (late Phanerozoic) when *m*Mg/Ca dropped to its lowest levels (∼0.5); such conditions are unfavorable for corals with aragonitic skeletons [5]. Significant amounts of calcite have already been identified in the aragonite skeletons of some scleractinian corals [6]. The origin of calcite in modern corals may stem from contamination due to the growth of other calcifying organisms, such as crustose coralline algae [7], microboring infilling [8], and diagenesis [9]. Bulk analyses using Raman microscopy and infrared spectroscopy have demonstrated that skeletons of newly settled *Acropora millepora* recruits are predominantly composed of aragonite, with no evidence of calcite [10]. The ability of scleractinians to produce aragonitic or calcitic skeletons seems to have been species-dependent during the Cretaceous Period [11]; an extant fossil of the Cretaceous scleractinian coral *Coelosmilia* sp. has an intact calcitic skeleton [11]. Relatively high amounts of calcite in the primary skeleton have been reported in modern adult corals in artificial Cretaceous seawater with a low *m*Mg/Ca value [12]. However, a study recently pointed out that these large quantities of calcite may have been deposited by secondary modes of calcification to fill pore spaces [1]. Thus, some doubt still exists as to whether scleractinian corals are able to make calcitic primary skeletons under low *m*Mg/Ca conditions.

Modern corals live in high *m*Mg/Ca seawater, which is favorable for aragonite precipitation; adult corals have an aragonitic skeleton that can act as a nucleus of crystallization for the developing skeleton. Therefore, additional information on skeletal mineralogy at the initial calcification stage of coral juveniles is needed to adequately assess the mineralization of scleractinian corals in calcite–aragonite seas. Here, we describe the formation of biologically controlled primary skeletons made of both calcite and aragonite in the modern scleractinian coral *Acropora tenuis* in seawater with a low *m*Mg/Ca.

## Methods

### Incubation of Juvenile Corals

Colonies of *A. tenuis* were collected on the northern reef patch off Sesoko Island, Okinawa [with permission from the Okinawa Prefectural Government (No. 21–14)], and kept in a running seawater tank at Sesoko Station, Tropical Biosphere Research Center, University of the Ryukyus (Okinawa, Japan) until natural spawning occurred. The seawater in the outdoor tank was pumped in from the ocean in front of the station. This water had a Mg/Ca ratio of 5.3, typical of modern seawater. Eggs and sperm were mixed for fertilization; they were rinsed twice in filtered (0.22 µm) seawater (FSW) ∼2 h after fertilization. Zygotes were then maintained in 2-L plastic vessels containing water at 26°C. Larval metamorphosis was induced by adding 2 µM Hym 248, a neuropeptide [13]. Larvae were settled and incubated in plastic dishes [14] containing different combinations of *m*Mg/Ca-manipulated seawater. Treatment levels comprised six different concentrations of Mg and Ca, which were prepared by mixing natural seawater and Mg-free water. We tested the following Mg/Ca ratios: 5.3, 2.7, 1.5, 1.0, 0.5, and 0. The Ca concentration across all treatments was held within a narrow range (9.8–10.5 mM). The Mg concentration ranged between 0 mM and 51.7 mM (Table 1). Mg-free solutions were prepared by adding NaOH to seawater until the pH reached 10.5, which caused Mg(OH)_2_ to precipitate. After precipitation, we adjusted the pH to 8.2 by adding HCl. We added NaHCO_3_ and CaCl_2_ to adjust total alkalinity and Ca concentrations. The concentrations of Ca and Mg were determined by atomic absorption spectrometry (Z-2000 series; Hitachi, Tokyo, Japan). The pH and total alkalinity were determined with a pH electrode (pH meter Orion 4-star; Thermo Scientific, Waltham, MA, USA) and the Gran plot procedure using a total alkalinity titrator (ATT-05; Kimoto, Tokyo, Japan), respectively. The pH of the manipulated seawater was in the range 8.1–8.3; total alkalinity was in the range 2.1–2.3 mmol kg^–1^. The natural pH and total alkalinity of Sesoko Island seawater were within the ranges 8.0–8.3 and 2.1–2.3 mmol kg^–1^, respectively. All manipulated seawater was passed through a 0.22-µm filter to preclude secondary calcification by contaminating organisms.

**Table 1:** Concentration of Ca and Mg, Mg/Ca ratio (*m*Mg/Ca), and CaCO_3_ structure formed by the scleractinian coral *Acropora tenuis*.

#	Ca (mmol l^−1^)	Mg (mmol l^−1^)	*m*Mg/Ca	CaCO_3_ structure	Growth rate (mg juvenile^−1^ month^−1^)
1	9.8	51.7	5.30	A	0.114±0.014^a^ (n = 18)
2	10.0	26.4	2.65	A	0.109±0.009^ab^ (n = 23)
3	10.3	15.0	1.46	A,C (<1%)	0.104±0.008^b^ (n = 31)
4	10.5	10.4	0.99	A,C (5.4±0.7%)	0.065±0.018^c^ (n = 14)
5	10.5	5.2	0.50	A,C (20.0±1.4%)	0.076±0.012^d^ (n = 27)
6	10.5	0.0	0.00	N	–

A: aragonite, C: calcite (wt %), N: no skeleton. Structures were confirmed by both Raman microscopy and X-ray diffraction (XRD). Content of calcite was determined by XRD (mean±se, n = 2, each). Growth rates not connected by same letter on the values are significantly different by Tukey-Kramer HSD test (*P*<0.05).

Each treatment was replicated sixfold in separate dishes. Juveniles at concentrations of 0.3–1.1 mL^–1^ (5–17 juveniles per 15-mL dish) were incubated at 26°C under a fluorescent lamp (20 µmol photons m^–2^ s^–1^ under a 12∶12 h light:dark cycle). After the symbiotic algae (*Symbiodinium* spp.) had been introduced to the juvenile corals, we continually recirculated manipulated seawater from 2-L reservoir tanks to the dishes. Juveniles were kept for 2 months under each of the experimental conditions. The 2-L reservoir tanks were recharged every 6 days. Concentrations of Ca and Mg, and total alkalinity were measured before and after replacement of the seawater supply. We measured a maximum total alkalinity change of 40 µmol kg^–1^ over the 6-day period between water replacements. Changes in Ca and Mg concentrations were not detected and the *m*Mg/Ca of seawater supplied did not change significantly.

After 2 months of incubation, the juveniles were killed. Their skeletons were treated with NaClO to remove tissue, and used for the estimation of growth rate and determination of crystal structure. Growth rates were calculated on a dry weight basis for 14–31 juveniles under each treatment level at the end of the incubation. We used one-way analysis of variance (ANOVA) and Tukey–Kramer post hoc honestly significant difference (HSD) multiple comparison tests (JMP 8; SAS Institute, Cary, NC, USA) to compare differences in growth rates among treatments.

### Determination of Crystalline Structure

A single juvenile skeleton from each treatment was used for Raman spectroscopy; 5–20 juvenile skeletons per treatment were used for X-ray diffraction (XRD) measurements. The crystal structure of CaCO_3_ was determined by laser Raman spectroscopy (LabRAM ARAMIS; Horiba, Tokyo, Japan; NRS-3100, JASCO, Tokyo, Japan). Each sample was scanned within a range of 40–2000 cm^–1^ using a 532-nm laser with a 300 line mm^–1^ grating. The spectra were compared with mineral-specific spectra of aragonite and calcite provided by the Raman spectral libraries (Horiba) and by Clode et al. [10]. Raman spectra comparing calcite with aragonite have overlapping peaks in the following regions: ∼154, ∼710, and ∼1084 cm^–1^. The spectra have mineral-specific peaks for calcite and aragonite at ∼281 and ∼204 cm^–1^, respectively [10]. To determine the planar distribution of calcite skeletons, we performed 15-µm step spectrum measurements from the base side of juvenile corals. For calcite and aragonite mapping, we classified the specific spectra of calcite and aragonite using the multivariate analysis provided in LabSpec6 software (Horiba). All spectra in the 15-µm step measurements were compared with reference spectra of aragonite and calcite to identify the crystal structure. The distribution areas of aragonite and calcite were also calculated with LabSpec6 software.

Scanning electron microscope (SEM) images corresponding to Raman mapping images were obtained by using a JCM-5000 NeoScope instrument (JEOL, Tokyo, Japan) at an accelerated voltage of 10 kV under high vacuum. For SEM imaging, we fixed the dry skeleton samples to a stage with adhesive tape and coated them for 1 min with platinum using an autofine ion coater (JEC-3000FC; JEOL).

We used XRD with a low background silicon holder (Ultima+; Rigaku, Tokyo, Japan) for quantitative determination of calcite content. Whole juvenile corals were powdered and analyzed by XRD. The content of calcite was quantified as the ratio between the peak intensities at 26.2° (aragonite) and 29.4°–29.5° (calcite). These ratios were compared with a standard calibration curve made by mixing pure aragonite and calcite powders (2%, 4%, 6%, 8%, 10%, and 30% calcite in aragonite). Coral reference material JCp-1 (Geological Survey of Japan, National Institute of Advanced Industrial Science and Technology) was used for the aragonite standard. Pure natural CaCO_3_ crystal was used as the calcite standard; this sample was obtained from epithermal calcite dug out from the Kushikino gold mine located in Kagoshima Prefecture, Japan.

## Results

### Growth Rates

The juveniles grew and formed CaCO_3_ skeletons over the 2 months under each treatment condition (other than treatment #6, in which the *m*Mg/Ca level tested was zero; Table 1). In treatment #6, the juveniles disintegrated and died within a few days of metamorphosis. The growth rate of juveniles was significantly slower in low *m*Mg/Ca water than growth under natural conditions (ANOVA, *F*_4, 107_ = 71.36, *P*<0.0001). Compared with rates measured under a *m*Mg/Ca treatment level of 5.3, juvenile growth rates decreased by 4%, 9%, 44%, and 34% under *m*Mg/Ca treatment levels of 2.7, 1.5, 1.0, and 0.5, respectively (Tukey’s HSD, *P*<0.05).

### Raman Spectral Mapping and SEM

**Figure 1:**
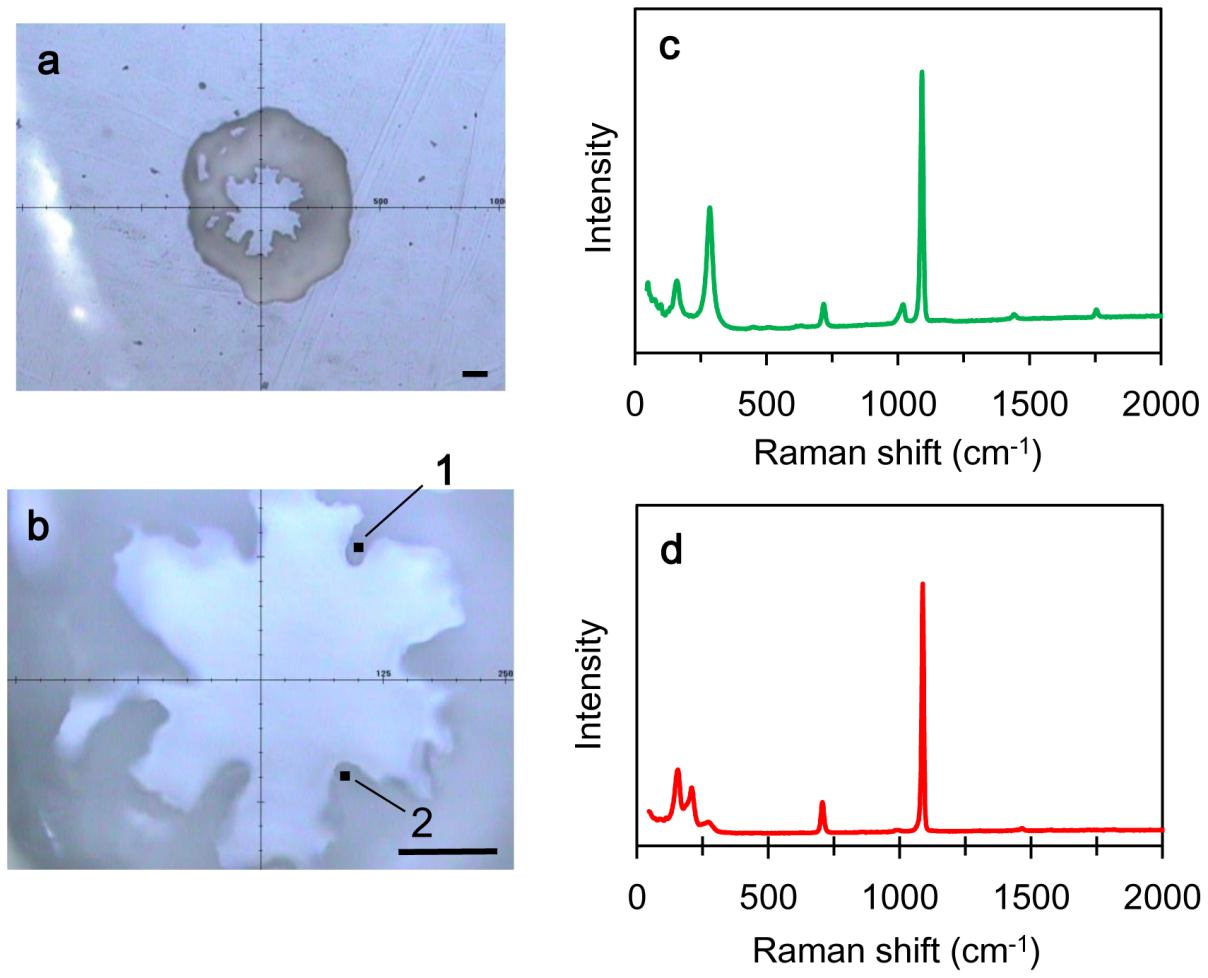
An observation image and Raman shift spectrum of a juvenile skeleton of *Acropora tenuis* grown in *m*Mg/Ca = 0.5 (#5, Table 1). (a) an observation image of a juvenile formed by the objective lens (×5); (b) an observation image of a juvenile formed by the objective lens (x20); (c) the specific Raman shift of calcite on dot #1 in Figure 1b; (d) the specific Raman shift of aragonite on dot #2 in Figure 1b. Scale bars are 100 µm.

**Figure 2:**
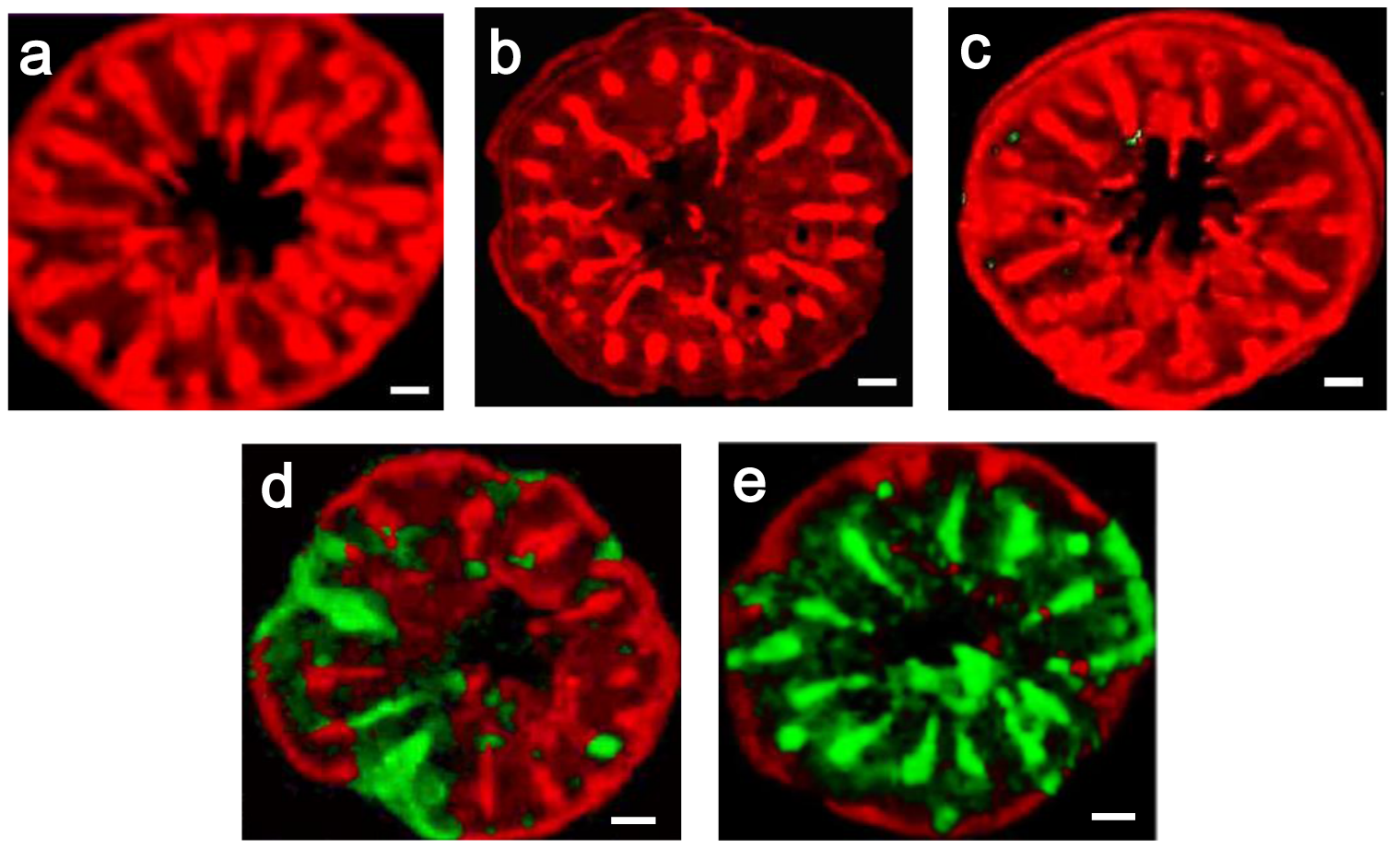
Raman mapping images of juvenile skeletons of *Acropora tenuis*. Scale bars are 100 µm. Red: aragonite, green: calcite. To classify Raman spectrum of calcite and aragonite, multivariate analysis was conducted. (a) *m*Mg/Ca = 5.3 (#1, Table 1); (b) *m*Mg/Ca = 2.7 (#2); (c) *m*Mg/Ca = 1.5 (#3); (d) *m*Mg/Ca = 1.0 (#4); (e) *m*Mg/Ca = 0.5 (#5).

**Figure 3:**
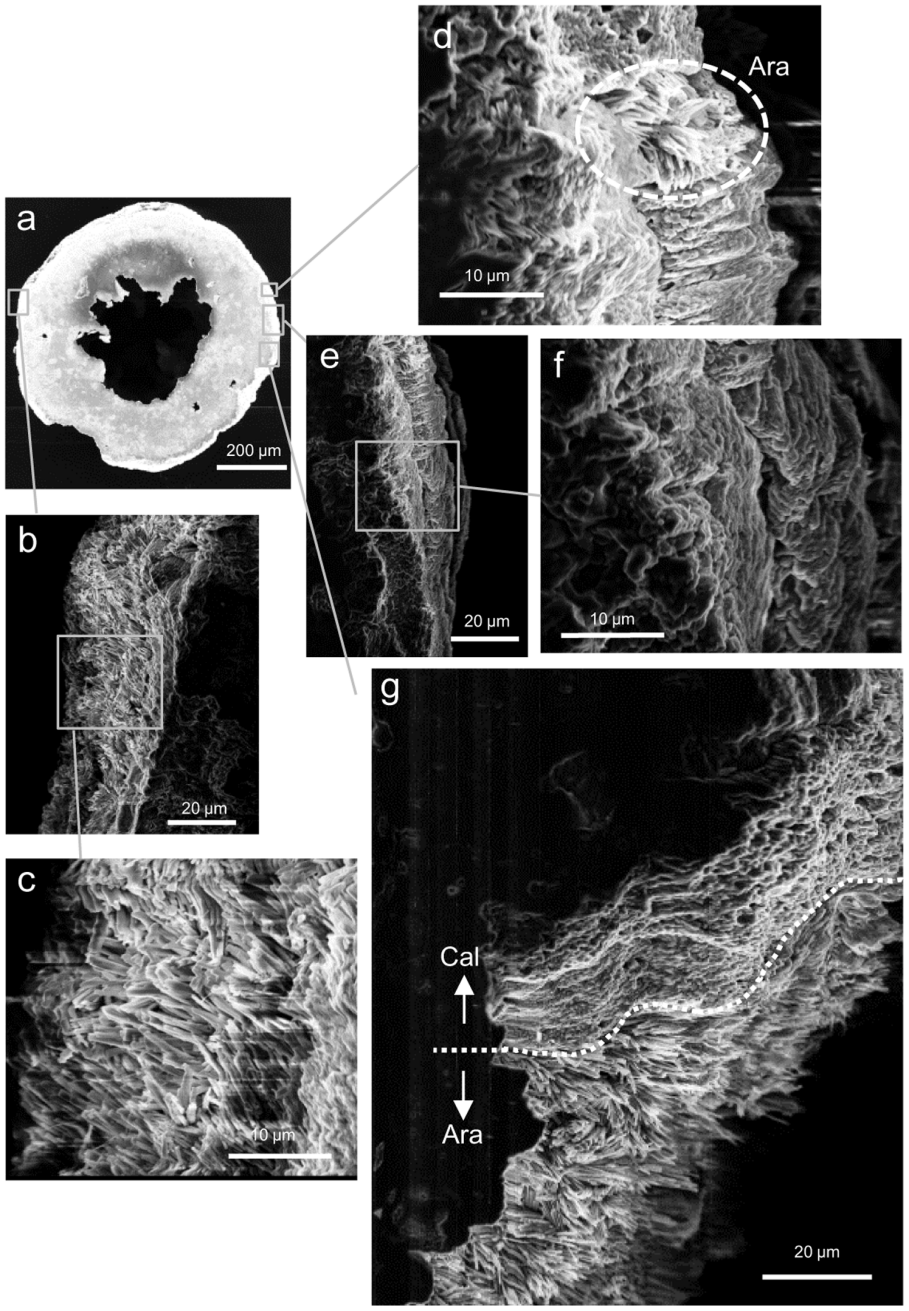
Scanning electron microscope (SEM) images corresponding to Figure 2e (*m*Mg/Ca = 0.5). (a) Whole juvenile skeleton (×100); (b) aragonite (×1000); (c) aragonite (×3000); (d) aragonite and calcite (×3000); (e) calcite (×1000); (f) calcite (×3000); (g) aragonite (underside of juvenile coral) and calcite (upper side of juvenile coral) (×1000). The crystal structures were determined by Raman spectroscopy (Figure 2e).

Figure 1 presents the Raman shift spectrum of a juvenile skeleton of *A. tenuis* raised at a *m*Mg/Ca treatment level of 0.5. Through comparisons with reference spectra, we were able to identify both calcite and aragonite spectral Raman shifts in this single juvenile skeleton (Figure 1c, d). For example, the round symbol #1 identifies a typical calcite spectrum that we found in the juvenile skeleton; peaks occurred at ∼159, ∼284, ∼717, and ∼1091 cm^–1^. Round symbol #2 identifies a spectrum for aragonite with peaks at ∼155, ∼ 208, ∼705, and ∼1087 cm^–1^. Figure 2 maps these specific Raman shifts for the whole juvenile skeleton, clearly demonstrating that juvenile corals incubated in seawater with low *m*Mg/Ca values (within the range 0.5–1.5) grew and made calcite skeletons (Figure 2c–e). As observed by Raman microscopy, the area of the calcitic skeleton increased with decreasing *m*Mg/Ca. No calcite was found in the skeletons under treatment conditions in which *m*Mg/Ca exceeded 2.6 (Figure 2a, b). At lower ratio values of 1.0 and 0.5, some parts of the septa were formed from calcitic skeleton material. Calcite distribution areas in the Raman mapping images made up 0.5%, 24.2±2.0% (mean ± SE, n = 3), and 48.3±5.4% (n = 2) of the total area in treatments with *m*Mg/Ca values of 1.5, 1.0, and 0.5, respectively. Figure 3 presents SEM images corresponding to the Raman spectral map in Figure 2e. In correspondence with the Raman spectrum, we observed different crystals of aragonite and calcite within a single juvenile skeleton.

### XRD Analysis

**Figure 4:**
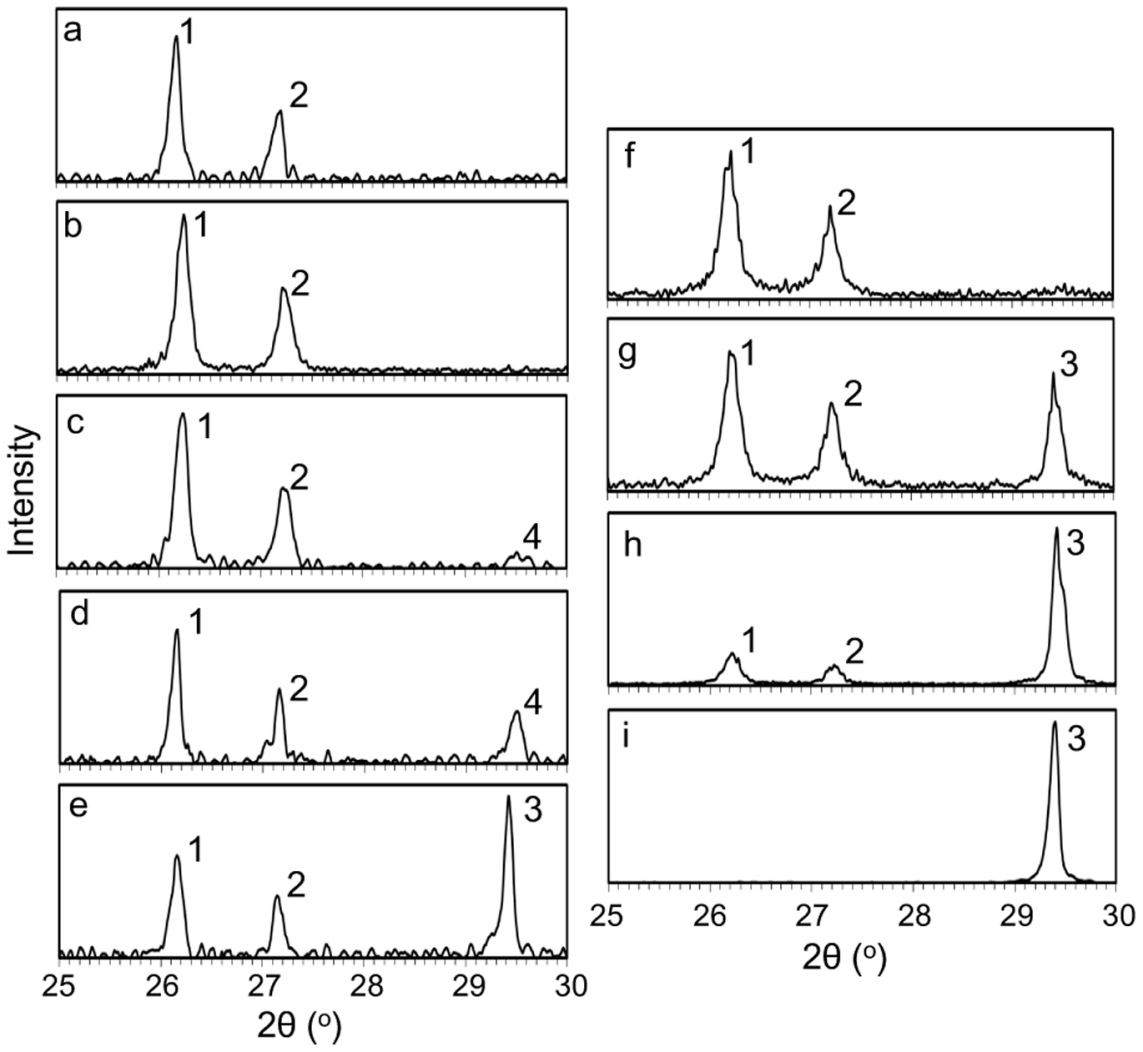
X-ray diffraction pattern of the juvenile skeletons of *Acropora tenuis* in manipulated *m*Mg/Ca seawater. (a) *m*Mg/Ca = 5.3 (#1, Table 1); (b) *m*Mg/Ca = 2.7 (#2); (c) *m*Mg/Ca = 1.5 (#3) calcite <1%; (d) *m*Mg/Ca = 1.0 (#4) calcite = 6.0%; (e) *m*Mg/Ca = 0.5 (#5) calcite = 21.8%; (f) 100% aragonite standard; (g) 90% aragonite and 10% calcite standard; (h) 70% aragonite and 30% calcite standard; (i) 100% calcite standard. The peaks numbers 1–4 of 2θ are 26.2, 27.2, 29.4, 29.5°, respectively.

Figure 4 presents the XRD pattern for single juvenile skeletons of *A. tenuis* grown in seawater with different *m*Mg/Ca values. Calcitic peaks of XRD (e.g., 2θ = 29.4°–29.5°) were found in treatments with low *m*Mg/Ca seawater (Figure 4c–e). Calcitic peak intensities increased with decreasing *m*Mg/Ca values in treatment seawater. Quantitative analysis using XRD showed that the majority of the skeleton was made up of aragonite across all treatments, with a maximum of 20% calcite in the low *m*Mg/Ca treatments. No calcite was observed under high *m*Mg/Ca conditions (>2.6, #1 and 2). We identified calcite under low *m*Mg/Ca conditions (<1.5, #3–5). Less than 1%, 5.4±0.9%, and 20.0±1.8% (mean ± SE, n = 2) of skeletal material was calcitic when *m*Mg/Ca values were 1.5, 1.0, and 0.5, respectively, in treatment seawater. By estimating the mole percentage of MgCO_3_ in calcite from the 2θ of the XRD [15], we determined that 2% low-Mg calcite (2θ = 29.5°) was produced when *m*Mg/Ca treatment values were in the range of 1.0–1.5 (#3, 4), whereas nearly pure calcite (2θ = 29.4°) was produced when the *m*Mg/Ca treatment value was 0.5 (#5). The calcite content was high under low *m*Mg/Ca treatment conditions, a trend similar to that for inorganic precipitation [16].

## Discussion

Our results showed that corals require Mg (minimum *m*Mg/Ca = 0.5) to survive in their early stages of development. A lack of Mg rapidly killed settling juveniles, likely because Mg is required for many biochemical reactions and enzymatic functions in living organisms [17]. In this study, the mass growth of each juvenile decreased by 44% and 34% when *m*Mg/Ca values in treatments were 1.0 and 0.5, respectively. Adult corals in low *m*Mg/Ca waters calcify at rates that are ∼60% lower than those in normal seawater [12]. Our results suggest that the effects of changed *m*Mg/Ca are less pronounced in juvenile corals than they are in adults. Thus, scleractinian corals have the potential to form skeletons in calcite seas. The strongest inhibition of growth was associated with the production of Mg calcite in the primary skeleton (when *m*Mg/Ca = 1.0). Inhibition of growth may therefore be caused by the instability of Mg calcite in comparison with both aragonite and calcite [18].

Calcitic septa were produced in low *m*Mg/Ca (<1.0) seawater. This is the first clear evidence that a modern scleractinian coral can form biogenic calcite septa at an early stage of development and is able to form a calcite skeleton. Nevertheless, the distribution of calcite was random and a majority of the skeleton was composed of aragonite. Modern scleractinian corals may possess calcite-forced organic matrix genes in addition to those for aragonite-forced organic matrices. Galaxin-related proteins [19], which are coral skeletal matrix proteins, have been found in *Acropora digitifera* [20]. Galaxin possesses small acidic domains since the Asx (aspartate plus asparagine) fraction in the galaxin is 9.7% [19]. The aspartic acid-rich proteins within the organic matrices of soft corals play a key role in calcitic crystal formation [21]. Organic matrices with acidic domains, including the Asx fraction, may facilitate formation of a calcite skeleton in scleractinian corals. Asx-rich candidate organic-matrix proteins have been identified in *Acropora* through direct isolation [20].

Estimates of calcite content by Raman mapping were higher than those provided by XRD. Our Raman mapping produced a planar image that did not show the whole skeleton. Both Raman microscopy and XRD indicated significant amounts of calcite in the coral skeletons. Raman mapping revealed the presence of calcite in many parts of the skeleton when *m*Mg/Ca treatment values were low. However, as indicated by XRD analysis, this widely distributed calcite remains quantitatively less important than aragonite in the skeleton. In addition, the density of aragonite might be higher than the density of calcite in modern corals. We consider the results of XRD (% weight of calcite) to be more quantitative for the whole skeleton than Raman planar mapping. In inorganic precipitation experiments, only calcite is formed when *m*Mg/Ca = 0.5 [16], [22]. The *Acropora* in this study constructed calcite skeletons when *m*Mg/Ca was less than 1.5. In both inorganic precipitation [16] and in coral incubation experiments, calcite was formed under low *m*Mg/Ca conditions. However, when we directly incubated corals in different *m*Mg/Ca value seawater, the major proportion of the skeleton was aragonite, even when *m*Mg/Ca values were low (i.e., 0.5). These results demonstrate the strength of biological control processes in the calcification of corals.

Thus, corals appear to preferentially form an aragonite skeleton, even in low *m*Mg/Ca water. In mollusk shells, soluble protein controls crystal phase switching between calcite and aragonite [23]. The coral skeletal organic matrix protein complex plays a dual role: (i) constituting an extracellular matrix that adheres to the newly formed skeleton and (ii) attaching calicoblastic cells to this skeleton-blanketing matrix [24]. Organic matrices and templates specify the nucleation of aragonite polymorphs in modern corals [25]. The skeletal organic matrix protein in Mediterranean coral, which has a natural aragonite-only skeleton, supports the inorganic precipitation of aragonite under low *m*Mg/Ca conditions [26]. The deposition of CaCO_3_ in coral skeletons is therefore strongly biologically controlled, even under low concentrations of Mg. In the coral skeletal formation process, seawater reaches the calcification region beneath the calicoblastic ectoderm and affects its skeletal mineralogy in a manner similar to abiotic precipitation [27]. However, the *m*Mg/Ca of seawater only slightly affected the chemistry of the coral calcification medium in this study, although the calcite content in the skeleton increased with decreasing *m*Mg/Ca in seawater. If corals are unable to control *m*Mg/Ca in the calcification medium, calcite precipitation must be facilitated under low *m*Mg/Ca conditions. Furthermore, the initial precipitation of calcite may change the *m*Mg/Ca (by consuming an excess of Ca), thereby allowing subsequent formation of aragonite; the distribution of aragonite within the coral may reflect movement of the calcifying fluid. Thus, we have demonstrated that the skeletal mineralization of modern scleractinian corals is determined not only by species and inorganic processes, but is also biologically controlled. This suggests that scleractinian corals have sustainable flexibility in skeletal growth, which enhances survival during aragonite–calcite sea changes. If *m*Mg/Ca changes (e.g., under calcite sea conditions), scleractinian corals may survive by using calcite to form parts of their skeletons.

Although shallow-water tropical reef organisms have been present through the last 500 million years, reef-building members of the Scleractinia first appeared in the fossil record in the middle Triassic (∼240 million years ago, Mya) [28]. Genomic analysis indicates a deep divergence of the coral *Acropora* and the sea anemone *Nematostella*, which cannot produce a calcified skeleton, ∼500 Mya [20]; this calculation suggests that corals existed in the planet’s oceans before the first fossils were laid down. However, the timing of the emergence of an ability to make calcified skeletons remains unknown. The absence of fossilized coral in the geological record is associated with periods of unfavorable conditions for calcification, such as ocean acidification or calcite seas [5]. Through the past 500 million years, CO_2_ in the atmosphere has varied, reaching values of over 4000 ppm [29], [30]. The naked coral hypothesis may explain the disappearance of corals from the fossil record [5]. Increased CO_2_ levels likely led to the decalcification of skeletons and the formation of naked corals able to survive and resume calcification when CO_2_ levels dropped [31]. However, the juvenile polyps of *Acropora* spp. formed skeletons under CO_2_ concentrations of ∼3000 ppm, concentrations [32] that corals have not experienced for at least 240 million years [29]; nevertheless growth rates decreased under these experimental conditions. The previous existence of calcite seas with low *m*Mg/Ca values has also been used to explain the reduced occurrence of corals in the fossil record [33]. However, we showed experimentally that corals can still calcify even with the lowest *m*Mg/Ca values. Therefore, modern *Acropora* is able to form skeletons under stresses resulting from ocean acidification [32] and the low *m*Mg/Ca values we independently tested in our study. However, the combined effects of low *m*Mg/Ca and high CO_2_, which have yet to be experimentally tested, might explain the disappearance of corals from the fossil record. Thus, periods with combined stresses imposed by simultaneously increased CO_2_ levels and decreased *m*Mg/Ca values may have greatly decreased the skeletal growth of scleractinians and resulted in corals losing their role as dominant reef builders [1], [30].

The ways in which marine calcifiers recovered from mass extinctions and the role played in this recovery by skeletal mineral compatibility with seawater have been discussed previously [34]. The hidden history of corals in the fossil record may represent a time when skeletonized scleractinians were rare or absent from the nearshore environments where fossil preservation potential is enhanced [35]. Under the combined stresses of high CO_2_ levels and low *m*Mg/Ca, corals may have rarely survived (even with small calcite skeletons) or may have moved to offshore environments. When the CO_2_ level dropped, corals may have constructed skeletons comprising mixtures of calcite and aragonite to extend their distributions in calcite seas after the mass extinction of dominant reef builders such as rudist bivalves [5]. Thus, scleractinian corals have shown uncommon adaptive capabilities in the face of global environmental change over long timescales and through mass extinction events, but no research has demonstrates whether scleractinians will be able to adapt to rapid environmental changes in the future.
